# Learning robotic pyeloplasty without simulators: an assessment of the learning curve in the early robotic era

**DOI:** 10.6061/clinics/2019/e777

**Published:** 2019-06-20

**Authors:** Mario F Chammas, Anuar I Mitre, Marco A Arap, Nicholas Hubert, Jacques Hubert

**Affiliations:** I Divisao de Urologia, Faculdade de Medicina FMUSP, Universidade de Sao Paulo, Sao Paulo, SP, BR; II Division of Urology, Centre Hospitalier Universitaire de Nancy, Nancy, France; III Divisao de Urologia, Hospital Sirio-Libanes, Sao Paulo, SP, BR

**Keywords:** Robotic-Assisted, Laparoscopy, Ureteropelvic Junction Obstruction, Education, Learning Curve

## Abstract

**OBJECTIVE:**

To analyze our experience and learning curve for robotic pyeloplasty during this robotic procedure.

**METHODS:**

Ninety-nine patients underwent 100 consecutive procedures. Cases were divided into 4 groups of 25 consecutive procedures to analyze the learning curve.

**RESULTS:**

The median anastomosis times were 50.0, 36.8, 34.2 and 29.0 minutes (*p*=0.137) in the sequential groups, respectively. The median operative times were 144.6, 119.2, 114.5 and 94.6 minutes, with a significant difference between groups 1 and 2 (*p*=0.015), 1 and 3 (*p*=0.002), 1 and 4 (*p*<0.001) and 2 and 4 (*p*=0.022). The mean hospital stay was 7.08, 4.76, 4.88 and 4.20 days, with a difference between groups 1 and 2 (*p*<0.001), 1 and 3 (*p*<0.001) and 1 and 4 (*p*<0.001). Clinical and radiological improvements were observed in 98.9% of patients. One patient presented with recurrent obstruction.

**CONCLUSIONS:**

Our results demonstrate a high success rate with low complication rates. A significant decrease in hospital stay and surgical time was evident after 25 cases.

## INTRODUCTION

Ureteropelvic junction obstruction (UPJO) still presents as a common congenital anomaly, usually preventing the normal urine flow from the kidney to the ureter. If not properly corrected, it can potentially lead to several conditions, including renal stone formation, recurrent urinary tract infections (UTIs) and eventually progressive renal deterioration 1-3.

The current management of UPJO includes both conservative and surgical options. Open pyeloplasty has been considered a gold standard for the treatment of this condition 4-6, but novel and less invasive methods progressively replaced the traditional approach 7.

Laparoscopic pyeloplasty is a viable and minimally invasive surgical technique, presenting favorable outcomes similar to those of the open technique. Therefore, laparoscopic pyeloplasty has replaced the traditional open approach and is considered a new gold standard for the surgical management of UPJO 8.

While laparoscopic pyeloplasty appears to be an attractive alternative, its learning curve may preclude the widespread use of this procedure. Vallancien et al. reported, after reviewing 1,311 procedures in a single center, that at least 50 complex surgeries should be performed over one year, with a frequency of at least one procedure per week to learn how to perform this procedure in a proper manner 9.

Robotic-assisted laparoscopic pyeloplasty (RALP) seems to be a reasonable substitute for pure laparoscopy, apparently reducing the procedurés learning curve 10. This approach has gained popularity, and several studies currently indicate RALP as a viable alternative with favorable outcomes 11-13.

Similar to other modern surgical techniques, robotic-assisted procedures require new training methods. In addition, the amount of time and experience necessary to learn the technique varies depending on the complexity of the procedure. The learning curve of robotic-assisted pyeloplasty has been studied before; however, most of these studies were aimed at the pediatric population 14,15. Whether the learning curve is reproducible for adults remains unknown. There is a paucity of studies addressing this issue, particularly in the early beginnings of the robotic era, when robotic simulators were not readily available. These studies could be useful in determining a minimal number of real cases necessary for a novice surgeon to master the basics of the procedure, which is important information that could potentially help to plan and design training and residency programs.

Here, we expected to analyze the learning curve for RALP in an adult population by reviewing the outcomes of the initial 100 cases performed by the same surgeon who had no previous exposure to robotic simulators.

## MATERIALS AND METHODS

From November 2001 to January 2007, 100 subsequent patients over 18 years old with UPJO underwent transperitoneal RALP. The appropriate ethics committee authorized the study. Informed consent was also obtained before the procedures.

Patients were ordered from number 1 to number 100 for this study in a consecutive manner. They were divided into 4 groups, with 25 patients in each. Group 1 comprised the first 25 patients submitted to the procedure, group 2 comprised patients 26 to 50, group 3 comprised patients 51 to 75 and group 4 comprised the final 25 patients. Patient features and operative results were recorded and included demographic characteristics, side of the obstruction, placement of a ureteral stent during or before pyeloplasty, operative time, estimated blood loss (EBL), suturing time, length of hospital stay (LOS) and surgical outcomes.

All procedures were performed by the same surgeon (JH), and the platform used was the da Vinci Surgical System from Intuitive Surgical (Sunnyvale, CA, USA). The main surgeon started his training in robotics one year before the clinical series began, when the da Vinci System was placed in an animal laboratory, and experimental surgeries were performed in pigs. During that period, fourteen pyeloplasties were completed using a transperitoneal laparoscopic approach with the robotic system 16. Because they were performed in an animal laboratory, those initial cases were not included for the learning curve count, which started with the first human procedure. Previously, with this training, the main surgeon had more than 20 years of experience in complex urological open surgeries but no prior exposure to laparoscopic surgery. No robotic simulators were available at our facility at that time, and no proctors were involved in the training process.

The protocol before RALP consisted of an intravenous urography (IVU) with furosemide and/or MAG-3 renography, which were performed to determine both renal function and the degree of obstruction. All patients were also evaluated with multidetector CT (MDCT) scanning to assess the renal anatomy and the presence of crossing vessels.

All surgical interventions were indicated due to the presence of symptoms, recurrent infection, loss of renal function or stone formation.

A dismembered pyeloplasty with a transabdominal approach, following the Anderson-Hynes' original technique, was performed in all patients as previously described by our group 17. The abdominal trocars were positioned as follows: two 10 mm metallic ports (Intuitive Surgical, USA) for the robotic arms were placed in the iliac fossa and subcostally, following the midclavicular line, a 12 mm port for the camera was placed on the para-umbilical line (Ethicon Endosurgery, USA), and an additional 12 mm accessory port (Ethicon Endosurgery, USA) was placed on the umbilicus to be used by the assistant surgeon.

During the initial part of the surgery, we typically used bipolar forceps and an electrocautery hook. After exposing the obstructed area, articulated scissors were used to transect the renal pelvis and excise the stenotic segment and the redundant pelvis if necessary. The ureter was then exposed, and its posterior side was spatulated. If urinary stones were present, they were typically removed with either the robotic arms or using a fibroscope that was introduced and navigated by the assistant surgeon. Two needle holders were then used for pelvi-ureteric anastomosis. A hydrophilic guidewire was then introduced in an antegrade manner through the assistant's port, and an 8 Fr JJ stent (Terumo Medical Corporation, USA) was left in place.

After completion of the anastomosis, the surgical site was retroperitonealized by closing the peritoneal layer with 4-0 absorbable running sutures.

During the initial postoperative period, analgesia usually included intravenous nonsteroidal anti-inflammatory drugs for the first one or two days, followed by oral medications thereafter, which were maintained during the entire hospital stay. Physical therapy and a liquid diet were started on the first postoperative day. A solid diet was introduced on the second day after the surgery.

The surgical parameters (operative time, suturing time, blood loss, intra- and postoperative complications, and conversions to open surgery) and patient outcomes were recorded, and a comparison between the groups was performed. A temporary bladder catheter was kept in place for at least the initial 48 hours. All patients were discharged with an indwelling double J ureteral catheter. The Clavien-Dindo classification of 2004 18 was used to classify the intra- and postoperative complications.

Clinical evaluation and stent removal were performed typically 4 to 6 weeks after the procedure. Imaging studies, including an IVU and/or a MAG-3 scan, were initially scheduled at 3 and 12 months postoperatively and then performed on an annual basis thereafter. The presence of either an obstruction of urine flow, confirmed by a radionuclide diuretic renogram with MAG-3, by a T ½ superior to 20 minutes, or by the recurrence of symptoms upon clinical follow-up indicated an unsuccessful outcome.

The learning curve was established following a comparison of the recorded variables between the groups. Statistical analysis was performed with the Minitab program (Minitab Statistical Software, Minitab Inc., USA). Variables with continuous values were compared using both the Kruskal-Wallis test and ANOVA. The equality of group variances was evaluated with the Bartlett test, and if significant differences between the groups were observed, then additional comparisons were investigated using the Bonferroni Test. Statistical significance was established at *p*<0.05.

## RESULTS

Table 1 presents the demographics and characteristics of the 99 patients (41 men and 58 women, with one bilateral procedure) who were admitted to our facility.

Twenty-one patients (8, 4, 4, and 5 in groups 1 to 4, respectively) had a ureteral stent placed during the preoperative period because of either significant pain (14 cases) or acute pyelonephritis (7 cases). In all of those patients, the catheter was maintained during the pyeloplasty and removed 4 to 6 weeks after the procedure in the same manner as in the other patients in the study.

Ninety-nine procedures were completed laparoscopically. One conversion to open surgery was necessary in one patient (in group 1) due to local fibrosis, difficult dissection and difficult exposure of the UPJO (20^th^ patient of the series).

**Figure 1:**
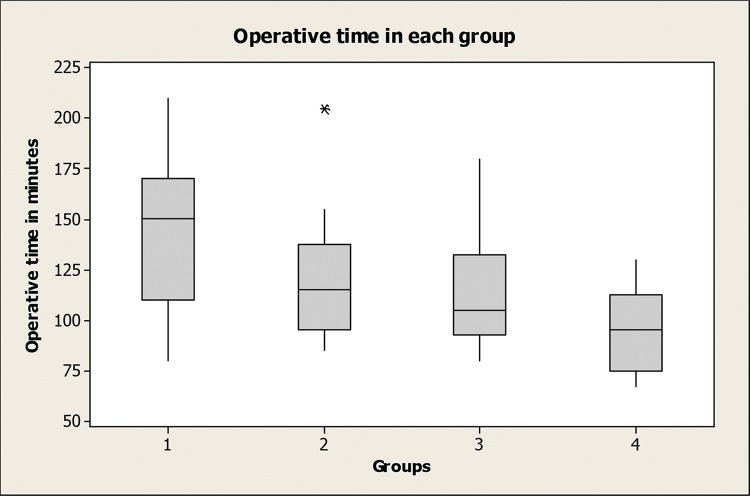
Operative time in each group.

**Figure 2:**
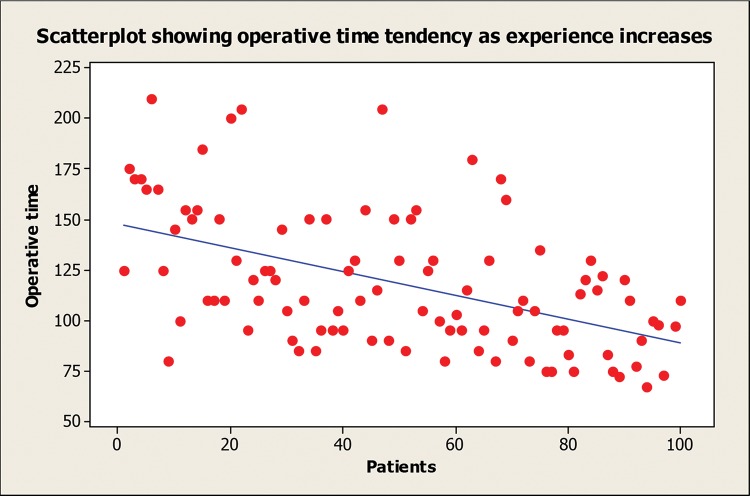
Analysis of the operative time.

The mean±standard deviation (range) of operative time for the whole series was 118±33.7 (67-210) minutes and 144.6±35.9, 119.2±28.6, 114.5±29.6 and 94.6±19.1 minutes for groups 1 to 4, respectively. A significant difference was observed between groups 1 and 2 (*p*=0.015), 1 and 3 (*p*=0.002), 1 and 4 (*p*<0.001) and 2 and 4 (*p*=0.022), showing a significant improvement in the operative time following the first 25 cases (Figure 1). A scatterplot analysis of the operative time showed a progressive decrease in surgical time as the surgeońs experience increased (Figure 2).

The median±standard deviation anastomosis times were 50.0±42.4, 36.8±11.6, 34.2±14.1 and 29.0±8.7 minutes for groups 1 to 4, respectively. Although a trend suggesting a decrease in the anastomosis time was evident, no statistical significance was demonstrated (*p*=0.137).

Surgical findings included 59 crossing vessels: 16, 12, 15 and 16 instances in groups 1 to 4, respectively; they were all detected preoperatively by the MDCT scans.

Sixteen patients (5, 4, 4 and 3 in groups 1 to 4, respectively) presented renal stones. These were managed using either robotic instruments (11 patients) or a flexible cystoscope introduced through the umbilical trocar (5 cases). All stones were removed successfully, and no residual calculi were present after the procedures.

When comparing the median±standard deviation operative times of the global series (118±33.7 minutes) with those 16 patients treated concomitantly for renal stones (119±37.4 minutes), no significant difference was demonstrated between the groups (*p*=0.88).

However, if a similar analysis is performed to evaluate the median±standard deviation operative time for the patients with previous UPJO management (157±33.7 minutes) and comparing the median±standard deviation operative times of the global series (118±33.7 minutes), a significant difference is present, demonstrating an extended operative time for the patients with a history of UPJO management (*p*<0.001).

The estimated blood loss was negligible for the whole series (<100 ml). No perioperative complications were recorded, except for one patient (group 1) who presented a small traumatic laceration of a largely hydronephrotic pelvis that occurred after the first trocar was placed. The lesion was noted as soon as the camera was introduced and was sutured primarily in the beginning of the case, and the surgery proceeded in an uneventful manner.

The robotic system malfunctioned in two cases (groups 2 and 4). In the group 2 patient, a sudden change in the color pattern of the auxiliary monitor appeared during the procedure (blue screen), but because the image of the surgeońs console showed no changes in the regular pattern, the procedure continued with no further issues. In group 4, one malfunction occurred in the surgeon's console imaging system, presenting an unexpected malfunction on the screen of the left eye (black screen). The image was reestablished shortly after the occurrence, and the procedure proceeded normally.

Significant postoperative complications (Clavien-Dindo Grade 3 or above) were noticed in only two patients in group 2 (Clavien grade 3b). Both presented a retrograde migration of the double J catheter.

One patient in group 1 and one in group 2 had an episode of pyelonephritis. Both patients were treated successfully with oral antibiotics.

A satisfactory degree of analgesia was obtained with NSAIDs in all patients, except for one patient in group 3 who required a morphine analog medication during the first postoperative day after the procedure. A visual analogue scale (range 1 to 10) for pain was utilized for all patients, and the median pain score was 2.4 on the first postoperative day and was similar in all groups (*p*=0.51).

Oral diet and light physical activity were restarted on the first postoperative day. The median±standard deviation hospital stay was 5.23±1.69 days, with 7.08±1.93, 4.76±0.83, 4.88±1.01 and 4.20±1.15 days for groups 1 to 4, respectively (*p*<0.001). A significant difference was present when comparing groups 1 *vs*. 2 (*p*<0.001), 1 *vs*. 3 (*p*<0.001) and 1 *vs*. 4 (*p*<0.001), demonstrating a reduction in the hospital stay after the first 25 cases.

The average follow-up was 50.6 months, with 60.2, 55.1, 47.9 and 39.9 months for groups 1 to 4, respectively. Seven percent of the patients (three from group 1, one from group 2, two from group 3 and one from group 4) were lost to follow-up and did not return for the first evaluation, which was 3 months after double-J catheter removal.

At 3 months postoperatively, a significant clinical improvement was present in all patients, except 6 patients (three from group 1, two from group 2 and one from group 4) presenting mild occasional flank pain. Those patients were completely asymptomatic after one year of follow-up.

All patients presented a radiological improvement in follow-up imaging studies, except two patients in group 1, who presented clinical improvement, but an IVU showed a delayed urinary excretion. A renal scan was performed for both patients, showing no obstruction at one year. One patient in group 3 presented with recurrent UPJO at a late follow-up (50 months). No other recurrences were present.

## DISCUSSION

Historically, open pyeloplasty was considered the gold-standard surgical treatment for UPJO patients 4-6. However, since the first report of a successful laparoscopic pyeloplasty In 1993, by Schuessler et al. 19, treatment options for these patients have evolved. By the end of the 20^th^ century, robot-assisted surgery debuted, establishing its role among other minimally invasive methods.

Robotic pyeloplasty was first suggested by Sung et al. in an experimental study in pigs using the Zeus System (Computer Motion, Goleta, CA, USA) 20. Later, a similar study using the da Vinci System was performed by Hubert et al. 16. In 2003, Yohannes and Burjonrappa performed the first human RALP 21. Currently, both pure laparoscopic 8,22,23 and RALP 24,25 approaches have been shown to be safe and effective and challenge open procedures as the gold standard for the treatment of UPJO 8.

Patel, reporting the outcomes of his initial 50 cases, published the first series of patients treated by RALP in the adult population. During his experience, he emphasized the short learning process related to the procedure 13. Later, our group compared our initial experience with Pateĺs, showing similar results, despite the presence of slight technical differences 17.

Mufarrij et al. also published a large, multicenter study including 140 cases with patients presenting both primary and secondary UPJO, concurrent stone extraction and solitary kidneys 26 with excellent results.

Several other groups have reported their experience with robotic pyeloplasty 24, and recently, Lucas et al. published a multi-institutional study with the Laparoscopic and Robotic Pyeloplasty Collaborative Group, aiming to compare laparoscopic and robotic pyeloplasty in a retrospective multicenter trial incorporating 865 cases from 15 centers 25. They concluded that both laparoscopic and robotic pyeloplasty were highly effective in treating UPJO, with similar excellent results.

In the era of minimally invasive surgery, the management of UPJO patients should be performed by a method that must be not only safe and effective but also easy to learn. The learning process of a surgical technique can be complex when new procedures and technologies are involved. Few studies have addressed the learning curve of robotic pyeloplasty, and the majority of them aimed at the pediatric population 14,15.

Currently, the definition of a learning curve is still lacking because many variables and outcomes such as positive margins, surgical times, surgical complications or hospital stay can be analyzed 15,27,28.

We believe that surgical time is an important factor when a learning curve is defined, but it must be assessed along with other parameters. In our study, we considered not only the operative time but also other parameters such as suturing time, LOS and the final clinical results when defining the learning curve. These outcomes were also previously discussed by other authors 15,27.

Furthermore, although a perfect frame for determining how learning curves should be approaches is still lacking, the current available studies that aimed to evaluate the learning process for robotic pyeloplasties have used the surgical time as their main parameter when stablishing their learning curves 14,15,29.

To determine how minimally invasive surgical learning curves are currently being assessed, a systematic search was recently performed by Harrysson et al., analyzing papers published from 1985 to 2012 in minimally invasive surgery that mention a learning curve 27. Five hundred and ninety-two studies were evaluated in this study, and time was the most commonly used proxy for the learning curve (86%). Intraoperative outcomes were used in 53% of the studies, postoperative outcomes in 52%, technical skills in 17%, and patient-oriented outcomes in 6% 27.

Another issue when evaluating the learning process of a new method is the ideal framework that should be used for this assessment. In our study, our patients were split into 4 groups of 25 each, and the mean duration of the procedure was compared among the groups. This pattern has been applied before 30. Furthermore, according to Harrysson et al. meta-analysis, a statistical analysis of the learning curves followed this similar framework in 70% of the papers they analyzed, where data were obtained after splitting the patients in consecutive groups and comparing the mean duration of the operation between them, with tests such as a students t-test, χ2 test or simple ANOVA 27.

When evaluating a learning curve, several other methods can also be applied. Sorensen et al. 14, for example, estimated the learning curve for robotic pyeloplasty in children after reviewing the records of 33 patients undergoing RALP and compared it with open repair cases performed by a senior surgeon. The learning process was considered successful when the operative times were consistently within one standard deviation of the average open pyeloplasty time performed by an experienced surgeon 14.

On the other hand, Ou et al. 29 have used the cumulative sum (CUSUM) method, a sequential analysis used for monitoring change detection, to assess the learning curve for laparoendoscopic single-site retroperitoneal pyeloplasty for UPJO.

In our series, a significant reduction in both operative time and hospital stay was evident after the first 25 cases, suggesting that a minimum of 25 cases seems to be a reasonable experience to master the basics of the procedure.

Similarly, Sorensen et al. suggested that 15 to 20 cases of RALP would be necessary to achieve equivalent outcomes and operative times compared to open pyeloplasty in the pediatric population 14. Conversely, Tasian et al. suggested that it would be necessary for a fellow to perform 37 casesto achieve the median operative time when an attending faculty was performing RALP in children 15.

As seen in previous reports, a drop in the operative time was demonstrated as the surgeon's experience increased. Our mean±standard deviation operative time was 118±33.7 minutes, slightly lower than described by Lucas et al. in a multicentric study with 465 robotic pyeloplasties and a mean operative time of 204 minutes 25. However, this result was equivalent to the data reported by Autorino et al. 24 in a recent meta-analysis evaluating multiple series of robotic laparoscopic pyeloplasty, showing mean operative times between 105 and 335 minutes.

From a practical standpoint, some lessons were learned after our initial 100 cases. One of them is that a surgical drain seems to be unnecessary after a few initial cases. It is unclear if a similar practice has been used by other groups 24. Another aspect observed in our study is that anatomically challenging cases can apparently be assessed by the same trocar configuration as in regular cases, which is in accordance with the current literature 31. In a similar fashion, no changes in trocar placement were considered necessary when obese patients were operated, differing from what was suggested by other authors 32-37.

The complication rate was low during the entire series. In our experience, only two patients in group 2 presented with Clavien-Dindo grade 3 or above complications (cranially migrated ureteral stents) that were removed uneventfully with a ureteroscope. A low incidence of complications was also reported by other series, ranging from 0 to 2% 11,25,26,.

In our study, diuretic renography and/or IVU showed unobstructed drainage in all patients at 12 months. One patient in group 3 presented a late recurrence of UPJO. Our results are similar to those of other laparoscopic robotic series, presenting excellent results even in the initial cases 11,13,17.

To our knowledge, no studies have previously examined the learning curve for RALP in the adult population, especially in the early beginning of the robotic surgery era, when surgical simulators were still not widely available.

Simulation programs for the robot-assisted surgical system da Vinci were created to teach novice surgeons the skills required for performing robot-assisted surgery. Presently, the most common simulators used for the da Vinci System are the SEP-Robot, dV-Trainer, RoSS, and the da Vinci Skills Simulator, which is a hardware pack loaded directly onto the actual da Vinci device^42^.

Some studies suggest that those simulators may improve the initial console training for apprentice surgeons 42. However, such devices have also been criticized due to their lack of fidelity and high costs 42. In our study, we aimed to analyze the learning curve for RALP in an environment where those simulators were not yet available; hence, they had no influence on the initial learning process of the surgical team.

To our knowledge, no studies have previously examined the learning curve for RALP in the adult population. However, our observations and results should be interpreted cautiously because the environment and experience of the surgeons may vary substantially. The single surgeon of this series (JH) had no particular experience in laparoscopic surgery before using the da Vinci System. The surgical team practiced the procedure with pigs for one year before starting this series 16.

Evaluating the learning curve for this procedure in an environment that was not previously exposed to surgical simulation forced us to analyze the data collected from the early beginning of our series, which started more than a decade ago. Although this could lead to a historical perspective of the procedure, no significant changes were applied to the technique used in the beginning of this series compared to patients currently operated at our service.

Analyzing this specific period of time is justified because the exposure to simulators could potentially affect the learning process in an uncontrolled manner 42 because they can be readily available and usually with no usage limits for the apprentice. Since this exposure would be difficult to measure, this could significantly affect an objective evaluation of the learning curve, leading to unreliable results.

On the other hand, our study has some limitations due to its retrospective nature, and although the main surgeon and operation room team were essentially the same, some slight changes in personnel between cases have occurred. Furthermore, it is critical that our results should be validated at other institutions before we can define the learning curve associated with adult robotic pyeloplasty with precision.

In summary, although further studies seem necessary to unify and identify the number of cases that are required to a novice surgeon to feel comfortable with the basic aspects of this technique, our series suggests that a significant decrease in hospital stay and surgical time could be achieved after an initial experience of 25 cases.

## AUTHOR CONTRIBUTIONS

Mario F. ChammasJr MR was responsible for the study design, collection and analysis of data, and manuscript preparation and review. Mitre AI was responsible for the study design, analysis of the data, and manuscript preparation and review. Arap MA was responsible for the statistical analysis and manuscript review. Hubert N was responsible for the collection and analysis of data, and manuscript review. Hubert J is the main surgeon in all cases, and participated in the data analysis, and manuscript preparation and review.

## Figures and Tables

**Table 1:** Patient demographics.

	Median±standard deviation (range)	Group 1	Group 2	Group 3	Group 4	*p*
Age (years)	38±18.3 (18-81)	40±17.7	41±20.0	38±19.2	33±16.4	0.51
BMI* (kg/m^2^)	23.3±4.5 (35.6-14.9)	23.3±4.2	24.0±4.6	22.8±4.1	23.0±5.1	0.79
Previous Abdominal Surgeries		7	10	10	8	
Previous UPJO treatment		3	1	2	2	
Horseshoe kidneys		1	1		1	

* Body mass index.

## References

[1] Halachmi S, Pillar G (2008). Congenital urological anomalies diagnosed in adulthood - management considerations. J Pediatr Urol.

[2] Anderson KR, Weiss RM (1996). Physiology and evaluation of ureteropelvic junction obstruction. J Eendourol.

[3] Kausik S, Segura JW (2003). Surgical management of ureteropelvic junction obstruction in adults. Int Braz J Urol.

[4] Arun N, Kekre NS, Nath V, Gopalakrishnan G (1997). Is open pyeloplasty still justified?. BR J Urol.

[5] O'Reilly PH, Brooman PJ, Mak S, Jones M, Pickup C, Atkinson C (2001). The long-term results of Anderson-Hynes pyeloplasty. BJU Int.

[6] Brooks JD, Kavoussi LR, Preminger GM, Schuessler WW, Moore RG (1995). Comparison of open and endourologic approaches to the obstructed ureteropelvic junction. Urology.

[7] Canes D, Berger A, Gettman MT, Desai MM (2008). Minimally invasive approaches to ureteropelvic junction obstruction. Urol Clin North Am.

[8] Moon DA, El-Shazly MA, Chang CM, Gianduzzo TR, Eden CG (2006). Laparoscopic pyeloplasty: evolution of a new gold standard. Urology.

[9] Vallancien G, Cathelineau X, Baumert H, Doublet JD, Guillonneau B (2002). Complications of transperitoneal laparoscopic surgery in urology: review of 1,311 procedures at a single center. J Urol.

[10] Yohannes P, Rotariu P, Pinto P, Smith AD, Lee BR (2002). Comparison of robotic versus laparoscopic skills: is there a difference in the learning curve?. Urology.

[11] Schwentner C, Pelzer A, Neururer R, Springer B, Horninger W, Bartsch G (2007). Robotic Anderson-Hynes pyeloplasty: 5-year experience of one centre. BJU Int.

[12] Peters CA (2008). Robotic pyeloplasty--the new standard of care?. J Urol.

[13] Patel V (2005). Robotic-assisted laparoscopic dismembered pyeloplasty. Urology.

[14] Sorensen MD, Delostrinos C, Johnson MH, Grady RW, Lendvay TS (2011). Comparison of the learning curve and outcomes of robotic assisted pediatric pyeloplasty. J Urol.

[15] Tasian GE, Wiebe DJ, Casale P (2013). Learning curve of robotic assisted pyeloplasty for pediatric urology fellows. J Urol.

[16] Hubert J, Feuillu B, Mangin P, Lobontiu A, Artis M, Villemot JP (2003). Laparoscopic computer-assisted pyeloplasty: the results of experimental surgery in pigs. BJU Int.

[17] Chammas MF, Hubert J, Patel VR (2007). Robotically assisted laparoscopic pyeloplasty: a transatlantic comparison of techniques and outcomes. BJU Int.

[18] Dindo D, Demartines N, Clavien PA (2004). Classification of surgical complications: a new proposal with evaluation in a cohort of 6336 patients and results of a survey. Ann Surg.

[19] Schuessler WW, Grune MT, Tecuanhuey LV, Preminger GM (1993). Laparoscopic dismembered pyeloplasty. J Urol.

[20] Sung GT, Gill IS, Hsu TH (1999). Robotic-assisted laparoscopic pyeloplasty: a pilot study. Urology.

[21] Yohannes P, Burjonrappa SC (2003). Rapid communication: laparoscopic Anderson-Hynes dismembered pyeloplasty using the da Vinci robot: technical considerations. J Endourol.

[22] Eden C, Gianduzzo T, Chang C, Thiruchelvam N, Jones A (2004). Extraperitoneal laparoscopic pyeloplasty for primary and secondary ureteropelvic junction obstruction. J Urol.

[23] Inagaki T, Rha KH, Ong AM, Kavoussi LR, Jarrett TW (2005). Laparoscopic pyeloplasty: current status. BJU Int.

[24] Autorino R, Eden C, El-Ghoneimi A, Guazzoni G, Buffi N, Peters CA (2014). Robot-assisted and laparoscopic repair of ureteropelvic junction obstruction: a systematic review and meta-analysis. Eur Urol.

[25] Lucas SM, Sundaram CP, Wolf JS, Leveillee RJ, Bird VG, Aziz M (2012). Factors that impact the outcome of minimally invasive pyeloplasty: results of the Multi-institutional Laparoscopic and Robotic Pyeloplasty Collaborative Group. J Urol.

[26] Mufarrij PW, Woods M, Shah OD, Palese MA, Berger AD, Thomas R (2008). Robotic dismembered pyeloplasty: a 6-year, multi-institutional experience. J Urol.

[27] Harrysson IJ, Cook J, Sirimanna P, Feldman LS, Darzi A, Aggarwal R (2014). Systematic review of learning curves for minimally invasive abdominal surgery: a review of the methodology of data collection, depiction of outcomes, and statistical analysis. Ann Surg.

[28] Mitre AI, Chammas MF, Rocha JE, Duarte RJ, Ebaid GX, Rocha FT (2013). Laparoscopic radical prostatectomy: the learning curve of a low volume surgeon. ScientificWorldJournal.

[29] Ou Z, Qi L, Yang J, Chen X, Cao Z, Zu X (2013). Preliminary experience and learning curve for laparoendoscopic single-site retroperitoneal pyeloplasty. J Laparoendosc Adv Surg Tech A.

[30] Joseph M, Phillips M, Rupp CC (2012). Single-incision laparoscopic cholecystectomy: a combined analysis of resident and attending learning curves at a single institution. Am Surg.

[31] Chammas MF, Mitre AI, Hubert N, Egrot C, Hubert J (2014). Robotic laparoscopic pyeloplasty. JSLS.

[32] Schwartz ML, Drew RL, Andersen JN (2003). Induction of pneumoperitoneum in morbidly obese patients. Obes Surg.

[33] Maker AV, Maker VK (2017). Techniques to perform robotic left adrenalectomy in the obese patient. Surg Endosc.

[34] Madan AK, Menachery S (2006). Safety and efficacy of initial trocar placement in morbidly obese patients. Arch Surg.

[35] Hackethal A, Brennan D, Rao A, Land R, Obermair A, Nicklin J (2015). Consideration for safe and effective gynaecological laparoscopy in the obese patient. Arch Gynecol Obstet.

[36] Bernante P, Foletto M, Toniato A (2008). Creation of pneumoperitoneum using a bladed optical trocar in morbidly obese patients: technique and results. Obes Surg.

[37] Altun H, Banli O, Karakoyun R, Boyuk A, Okuducu M, Onur E (2010). Direct trocar insertion technique for initial access in morbid obesity surgery: technique and results. Surg Laparosc Endosc Percutan Tech.

[38] Singh I, Hemal AK (2010). Robot-assisted pyeloplasty: review of the current literature, technique and outcome. Can J Urol.

[39] Yanke BV, Lallas CD, Pagnani C, McGinnis DE, Bagley DH (2008). The minimally invasive treatment of ureteropelvic junction obstruction: a review of our experience during the last decade. J Urol.

[40] Gupta NP, Nayyar R, Hemal AK, Mukherjee S, Kumar R, Dogra PN (2010). Outcome analysis of robotic pyeloplasty: a large single-centre experience. BJU Int.

[41] Palese MA, Stifelman MD, Munver R, Sosa RE, Philipps CK, Dinlenc C (2005). Robot-assisted laparoscopic dismembered pyeloplasty: a combined experience. J Endourol.

[42] Lyons C, Goldfarb D, Jones SL, Badhiwala N, Miles B, Link R (2013). Which skills really matter? proving face, content, and construct validity for a commercial robotic simulator. Surg Endosc.

